# Commentary on the T1D Exchange Quality Improvement Collaborative Learning Session November 2025 Abstracts

**DOI:** 10.1111/1753-0407.70214

**Published:** 2026-04-13

**Authors:** Stephanie S. Crossen, Nicole Rioles, Claire Rainey, Halis K. Akturk

**Affiliations:** ^1^ Division of Pediatric Endocrinology University of California Davis School of Medicine Sacramento CA USA; ^2^ T1D Exchange Boston MA USA; ^3^ Barbara Davis Center for Diabetes, Adult Clinic, University of Colorado Aurora Colorado USA

**Keywords:** health equity, learning health system, quality improvement, type 1 diabetes mellitus, type 2 diabetes mellitus

The T1D Exchange Quality Improvement Collaborative (T1DX‐QI) is a network of 63 adult and pediatric diabetes centers [1, 2] which collectively care for more than 150,000 people with diabetes (PWD) across the United States. Member centers map and report their data on diabetes care and outcomes and engage in quality improvement projects with the shared goal of improving health outcomes for all PWD. Each November, the group hosts its annual Learning Session to share lessons learned and facilitate ongoing collaboration on key topics of interest. This year’s Learning Session touched on a broad range of topics, from diabetes technology to mental health, sick day protocols to transitions of care, and reflected new emphases within the collaborative on screening and early identification of type 1 diabetes, and on optimizing care for type 2 diabetes in pediatric populations.

Over a dozen abstracts focused on improving timing or equity in the adoption of diabetes technology. Several centers initially deployed surveys to patients [3, 4] or clinicians [5, 6] to target their technology‐related QI efforts within a complex system. Clinicians involved in a multi‐center QI project to accelerate automated insulin delivery (AID) adoption identified insurance coverage, standardized clinic processes, patient and clinician education about AID, and communication with pharmacies and DME suppliers as key drivers and potential barriers to early AID access [7, 8]. QI initiatives focused on continuous glucose monitoring (CGM), in turn, cited the importance of addressing clinician bias, improving device accessibility, and developing standardized education [9, 10]. Many centers described multi‐pronged interventions that expanded access to multidisciplinary visits or classes to successfully reduce wait times [11, 12, 13] or increase overall uptake of technology [9, 14, 15].

Transition of care from pediatric to adult endocrinology also continued to be a focus at this year's learning session. Several projects found that the success of new electronic health record (EHR) or educational tools to aid in transition‐of‐care planning hinged on clinician time and awareness [16, 17] and that even with standardized questionnaires, topics like substance use, reproductive health, and insurance navigation were rarely discussed by pediatric clinicians [18]. However, embedded EHR transition tools were used successfully at several centers [19, 20] and a multidisciplinary approach to transition coordination was found to improve the documentation of transition plans for adolescents with diabetes [19] and to reduce the wait time between pediatric and adult care [21].

Screening for mental health concerns, social drivers of health, and comorbid medical conditions also featured prominently. Several abstracts described efforts to improve screening for depression and/or diabetes distress [22, 23, 24], disordered eating behaviors [25], or fear of hypoglycemia [26], while others focused on improving dietitian access [27], resources to address food insecurity [28], evaluating health literacy [29], or screening and education about exercise [30]. One center surveyed caregivers of youth with T1D on the role of social media and found that 49% of respondents followed a health influencer, with 25% of these changing their children's diet in response to this influence [31]. Abstracts focused on screening for comorbid conditions found that changes to in‐visit clinic processes and the addition of new equipment were most effective at increasing screening for nephropathy [32], retinopathy [33], peripheral arterial disease [34], and peripheral neuropathy [35].

Many T1DX‐QI centers reported this year on their innovative programs to identify and assist patients with elevated HbA1c or frequent diabetes related hospitalizations, and to improve safety in hospital and home settings. Two centers developed diabetes ketoacidosis (DKA) risk scores or prediction models [36, 37], while other centers showed that intensive contact from care team members after DKA events could reduce recurrence [38, 39]. Other high‐risk care models identified patients with elevated hemoglobin A1c (HbA1c) and demonstrated improvements in HbA1c or time‐in‐range after additional team‐based care [40] or remote monitoring of CGM data [41], respectively. Two centers reported on projects to improve insulin administration and safety in hospital settings [42, 43], and three abstracts focused on management of sick days or infusion set failures with pump/AID systems, demonstrating the importance of patient and clinician education [44, 45], and the broad consensus about sick‐day management with AID despite lack of published guidelines [46].

Two topics that received heightened attention this year were screening for early detection of T1D and management of pediatric type 2 diabetes (T2D). Early stage T1D screening programs found widely varying rates of stage 1 + 2 positivity depending on the base population screened [47, 48]. However, there was broad consensus that successful screening programs require dedicated clinical time, specific educational materials, clear clinical protocols, and new administrative workflows and EHR tools [47, 48, 49]. Responding to the urgency of rising incidence [50] and high complication rates [51] for youth‐onset T2D, centers also reported on a variety of projects to improve care for this condition. These ranged from evaluation of CGM receptivity among patients and families [52] to characterization of GLP‐1 receptor agonist prescribing patterns by clinicians [53], and the use of supplemental phone outreach to improve clinic return rates [54].

In summary, the 2025 T1DX‐QI Collaborative Learning Session highlighted exciting work being done by centers across a variety of domains related to improving care for PWD. The data, strategies, and tools outlined in the conference abstracts are collectively advancing the quality of care provided nationally and can be utilized broadly by health teams and systems dedicated to this common goal.

## Ethics Statement

The work of the collaborative is under a central IRB that was determined to be non‐human subjects research under the review of WCG.

## Conflicts of Interest

The authors declare no conflicts of interest.

## Data Availability

The commentary reflects data from the T1D Exchange Quality Improvement data set.
